# 3D-Printed Poly(ester urethane)/Poly(3-hydroxybutyrate-co-3-hydroxyvalerate)/Bioglass Scaffolds for Tissue Engineering Applications

**DOI:** 10.3390/polym16233355

**Published:** 2024-11-29

**Authors:** Nayla J. Lores, Beatriz Aráoz, Xavier Hung, Mariano H. Talou, Aldo R. Boccaccini, Gustavo A. Abraham, Élida B. Hermida, Pablo C. Caracciolo

**Affiliations:** 1 Research Institute for Materials Science and Technology, INTEMA (UNMdP-CONICET), Av. Colón 10850, Mar del Plata B7606BWV, Argentina; naylalores@gmail.com (N.J.L.); xhung@fi.mdp.edu.ar (X.H.); mtalou@fi.mdp.edu.ar (M.H.T.); gabraham@fi.mdp.edu.ar (G.A.A.); 2 Instituto de Tecnologías Emergentes y Ciencias Aplicadas (ITECA), Escuela de Ciencia y Tecnología (ECyT), Universidad Nacional de San Martín, CONICET, San Martín, Buenos Aires B1650, Argentina; baraoz@unsam.edu.ar (B.A.); ehermida@unsam.edu.ar (É.B.H.); 3 Institute of Biomaterials, Department of Materials Science and Engineering, University of Erlangen-Nuremberg, 91058 Erlangen, Germany; aldo.boccaccini@fau.de

**Keywords:** segmented polyurethanes, bioglass, poly(3-hydroxybutyrate-co-3-hydroxyvalerate), filament preparation, additive manufacturing

## Abstract

Biodegradable polymers and bioceramics give rise to composite structures that serve as scaffolds to promote tissue regeneration. The current research explores the preparation of biodegradable filaments for additive manufacturing. Bioresorbable segmented poly(ester urethanes) (SPEUs) are easily printable elastomers but lack bioactivity and present low elastic modulus, making them unsuitable for applications such as bone tissue engineering. Strategies such as blending and composite filament production still constitute an important challenge in addressing SPEU limitations. In this work, SPEU-poly(3-hydroxybutyrate-co-3-hydroxyvalerate) (PHBV) blends and SPEU-PHBV-Bioglass 45S5^®^ (BG) composite materials were processed into filaments and 3D structures. A comprehensive characterization of their morphology and thermal and mechanical properties is presented. The production of 3D structures based on SPEU-PHBV with excellent dimensional precision was achieved. Although SPEU-PHBV-BG printed structures showed some defects associated with the printing process, the physicochemical, thermal, and mechanical properties of these materials hold promise. The blend composition, BG content and particle size, processing parameters, and blending techniques were carefully managed to ensure that the mechanical behavior of the material remained under control. The incorporation of PHBV in SPEU-PHBV at 70:30 *w*/*w* and BG (5 wt%) acted as reinforcement, enhancing both the elastic modulus of the filaments and the compressive mechanical behavior of the 3D matrices. The compressive stress of the printed scaffold was found to be 1.48 ± 0.13 MPa, which is optimal for tissues such as human proximal tibial trabecular bone. Therefore, these materials show potential for use in the design and manufacture of customized structures for bone tissue engineering.

## 1. Introduction

From a materials science perspective, composite systems offer an exceedingly attractive pathway to design structures with tailored mechanical, physicochemical, and/or biological properties, thus catering to unique application requirements. Biodegradable polymers and bioceramics give rise to a wide range of composite structures that serve as scaffolds to promote tissue regeneration processes [1]. A crucial aspect of the success of 3D matrices in tissue engineering is that their structure and structural properties must closely resemble those of the tissue being regenerated [2]. Notably, biodegradable polymers hold considerable promise due to their ability to degrade into non-toxic byproducts, resulting in the complete elimination of macromolecules [1]. Additionally, their potential to enhance cell adhesion, migration, proliferation, and differentiation is noteworthy [1,2,3,4]. Typically, bioceramics are incorporated as fillers or coatings into the polymeric matrix to increase its strength and stiffness while also inducing bioactivity [1,2,5,6]. Like most ceramic materials, bioceramics have the drawback of exhibiting low fracture toughness, which limits their use in load-bearing applications [2,5]. Furthermore, their high rigidity can restrict their use in applications unrelated to bone tissue, for instance, in soft tissues where a much lower elastic modulus is required [5]. To overcome these issues, combining bioceramics with polymers to produce composite materials represents a strategy that leverages the properties of both materials to mimic the mechanical loads that specific tissues will experience in vivo [5].

Currently, numerous research groups are actively exploring the utilization of biodegradable and biocompatible polymeric filaments to create customized 3D matrices for various applications [7,8,9,10,11]. Particularly, the potential of combining polymer/ceramic biomaterials holds promise for specific applications, such as bone tissue engineering [6,10,11]. Hutmacher and colleagues were pioneers in this field, achieving the first 3D-printed structure from polycaprolactone (PCL)/HA composites by fused deposition modeling (FDM) [12]. Furthermore, FDM-printed matrices for bone tissue engineering have been developed using biopolymer/bioceramic composites [13,14,15,16,17].

Interest in additive manufacturing of biodegradable elastomers has been rapidly growing due to their wide range of applications in tissue engineering [10,11,18]. While this method promises faster and more precise fabrication compared with traditional production [11], additive manufacturing of elastomers—specifically segmented poly(ester urethanes) (SPEUs)—remains a challenge due to their unique properties and the limited selection for processing by FDM [10,11,18,19]. Particularly, SPEUs present high versatility due to their highly variable chemistry that can undergo phase separation due to the immiscibility of hard and soft segments. Thus, through the appropriate selection of monomers, SPEUs with a wide range of physicochemical, mechanical, and biological properties can be synthesized. However, they lack bioactivity and exhibit a low elastic modulus for applications such as bone tissue engineering [10,11].

On the other hand, poly(3-hydroxybutyrate-co-3-hydroxyvalerate) (PHBV) is a compelling biocompatible polyester of substantial interest across diverse biomedical applications. PHBV is an aliphatic polyhydroxyalkanoate (PHA), thermoplastic, and semicrystalline polymer with a high elastic modulus. Additionally, it displays biodegradability with non-cytotoxic degradation byproducts, as well as antibacterial properties, making it a favorable alternative for replacing conventional polymers and addressing environmental concerns [20,21,22,23]. Its degradation period is approximately 2 years, which is sufficient to promote the formation of new bone [21,24]. Blends of PHA with synthetic biodegradable polymers have been used to fabricate 3D matrices intended for tissue engineering applications [20,25,26,27]. Therefore, the blending of SPEU with PHBV holds promise for the production of elastomeric filaments that are both easy to process and possess a high elastic modulus, potentially useful in biomedical applications.

Similarly, polymer/bioceramic composites are being extensively explored. Bioglass 45S5^®^ (BG), a bioactive glass belonging to the silicate bioactive glass family (45% SiO_2_-24.5% Na_2_O-24.5% CaO-6% P_2_O_5_ *w*/*w*), occupies a crucial role in the realm of biomimetic materials, being the first entirely synthetic material to exhibit a remarkable ability in fostering a rapid and enduring direct chemical bond with bone tissue [21,28,29]. BG is characterized by its biocompatibility, osteoconductivity, non-toxicity, and lack of inflammation, creating an ideal environment for cellular colonization and proliferation and promoting enhanced differentiation of human osteoblasts to form new bone [3,6,21,30]. Conversely, its brittleness and low fracture toughness have prompted the development of numerous formulations combining it with synthetic biodegradable polyesters or natural polymers to generate 3D matrices with improved mechanical performance and biological behavior for bone tissue engineering [1,30] and soft tissue regeneration [29,31,32]. BG can enhance vascularization through the dissolution of biologically active ions, thus potentially also playing an important role in soft tissue repair (e.g., wound healing) [33,34,35,36,37].

The primary objective of this research was to assess the ability of SPEU/PHBV blends and polymer/BG composite materials to produce filaments and 3D-printed structures designed for tissue engineering applications, particularly focusing on bone tissue engineering. While other researchers have employed BG with polyesters for bone regeneration [17,31] and PHBV with other natural polymers for various applications [22], the specific combination of SPEU, PHBV, and BG to create 3D-printed scaffolds has not been reported to the best of our knowledge. The study includes a comprehensive characterization of raw materials, filaments, and 3D matrices concerning their physicochemical, morphological, thermal, and mechanical properties. This analysis, more in-depth than most presented in the literature, provides valuable insights into structure-property relationships, potentially contributing to the overall novelty of the work. While our study primarily targets bone tissue engineering, the broader potential of BG for soft tissue applications [32] hints at a future research direction for this unique composite.

## 2. Materials and Methods

### 2.1. Materials

Bioresorbable aliphatic segmented poly(ester urethane) (SPEU) containing 50% *w*/*w* hard segments was synthesized by a classic two-step polymerization method, according to our previous work [38]. PHBV copolymer (ENMAT Y1000 with a 1.5% mol content of valerate) was purchased from TianAn Biologic Materials Co., Ltd., Ningbo, China. Bioglass 45S5^®^ (BG) in the form of a powder with a particle size of approximately 5 μm was acquired from Schott AG (Schott AG, Mainz, Germany). PHBV and BG were used as received.

### 2.2. Filament Fabrication

SPEU and PHBV were mixed using an IKA A11 basic mill with liquid nitrogen to prevent thermal degradation. The obtained powders were processed into filaments using a parallel twin-screw mini-extruder (Thermo Fisher Scientific Process 11, Waltham, MA, USA). SPEU-PHBV filaments were prepared at a ratio of 70:30 *w*/*w*. Polymer/BG composite was prepared by incorporating 5 wt% of BG into the 70:30 *w*/*w* polymer blend and named SPEU-PHBV-BG.

The extrusion parameters were set according to our previous work [39], with screw speeds ranging from 50 to 75 rpm. The produced filaments were pulled out and spooled by a homemade rolling machine. As SPEU represents the major component of the blends, its extrusion temperature profile was used as a reference [38], considering that PHBV thermal degradation starts above 160 °C. Additionally, the last three heating zones of the extruder were set at slightly lower temperatures to increase the cooling speed and facilitate the solidification of the extruded filaments. Subsequently, the produced filaments were vacuum-sealed and stored with silica gel in a desiccator at room temperature until their use.

### 2.3. Additive Manufacturing by FDM

Structures were fabricated by FDM from filaments heated and melted within a 400 μm diameter extrusion nozzle. Cylindrical structures were designed with a pore size of 350 µm, a rectilinear pattern, and an interconnected porosity of approximately 50% based on CAD models (3D Builder v20.0.4.0 and Rhinoceros^®^ v6.1 software). For compression tests, the dimensions of the 3D scaffolds were 15 mm in diameter and 7.5 mm in height (21 layers). A height-to-diameter ratio lower than 1 was set to avoid potential buckling. Structures with 2 layers were also designed and printed.

### 2.4. Filament and Printed Structures Characterization

The physicochemical and morphological characterization of filaments and printed structures were performed as detailed in our previous work [39] (Supplementary Material). Analyses included Fourier transform infrared (FTIR) spectroscopy, differential scanning calorimetry (DSC), thermogravimetric analysis (TGA), water contact angle measurements, apparent porosity determination of scaffolds, surface morphology observation, and mechanical testing of filaments and 3D-printed scaffolds (according to ASTM D695 [40]).

## 3. Results and Discussion

### 3.1. Composite Filament Fabrication

While establishing the processing conditions for SPEU material is less challenging than for PHBV, SPEU lacks bioactivity and exhibits a low elastic modulus, which limits its application in areas such as bone tissue engineering. Conversely, although PHBV possesses these desirable properties, its inherent stiffness and brittleness make the production of pure copolymer filaments and their subsequent processing into 3D structures more challenging. Consequently, the utilization of polymer blends emerges as a promising strategy.

SPEU-PHBV 70:30 blend and SPEU-PHBV-BG composite materials were successfully employed in the fabrication of filaments suitable for processing into 3D structures without the need for additional additives. These systems enabled the production of continuous filaments that could be smoothly wound onto the winding machine, offering a simpler process compared with pure PHBV [21] and even blends with an SPEU content below 30% *w*/*w*. The precise control of extrusion speed and temperature profiles ensured the consistent production of filaments with uniform diameters. The use of a 1.5 mm nozzle resulted in an average filament diameter of 1.49 mm for all samples. Polymer blend composition, BG characteristics (content, particle size and distribution), processing parameters, and blending techniques were carefully managed to ensure that the mechanical behavior of the printed material remained under control. Such controlled methodology provides a reliable pathway for achieving reproducible mechanical performance in the synthesized composites.

#### 3.1.1. Morphological Characterization

**Figure 1:**
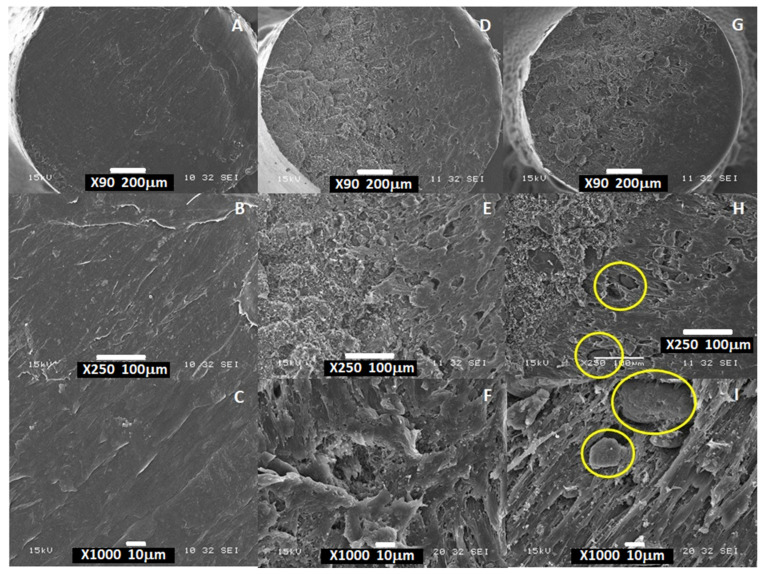
SEM micrographs of the cross-sectional views of the filaments: SPEU (**A**–**C**), SPEU-PHBV (**D**–**F**), and SPEU-PHBV-BG ((**G**,**H**,**I**), BG microparticles in yellow circles) (90×, 250×, and 1000×, respectively).

**Figure 2:**
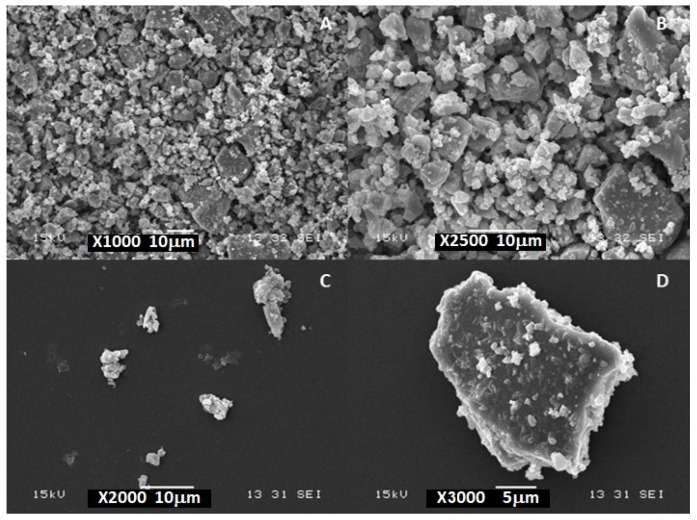
SEM micrographs of BG particles: (**A**–**D**) (1000×, 2000×, 2500×, and 3000×, respectively).

The microstructure of SPEU-based filaments was studied by examining their cross-sectional morphology. Figure 1A–C show the SEM micrographs of plain SPEU filaments. A smooth and homogeneous surface, typical of semi-crystalline polymers, was observed. However, the SPEU-PHBV and SPEU-PHBV-BG filaments displayed porous surfaces (Figure 1D–F and G–I, respectively), likely attributed to the extrusion process conditions and the narrow temperature range for processing PHBV [21,22]. Furthermore, the analysis for SPEU-PHBV filaments revealed a continuous phase of SPEU with PHBV dispersed in the form of spheres, suggesting the immiscibility of these polymers (Figure S1A,C in the Supplementary Material). A close examination of the fractured surfaces of SPEU-PHBV-BG (Figure S1C) revealed that the round-shaped PHBV particles had a mean diameter of approximately 800 μm with a rough surface. Similar patterns were observed in other polymeric blends containing PHBV [41]. In the case of SPEU-PHBV-BG filaments, BG microparticles were primarily located within the filaments, exhibiting a random distribution. These microparticles appeared as a separate phase with minimal interaction with the surrounding polymeric matrix (Figure 1H,I and Figure S1B). Figure 2 displays the SEM micrographs of BG microparticles, in which despite the manufacturer’s reported average size of 5 μm, a broader size distribution is observed.

In addition, the increased porosity was correlated with a decrease in filament density, as expected (density values: SPEU = 1.24 g cm^−3^; SPEU-PHBV = 1.07 g cm^−3^; and SPEU-PHBV-BG = 0.87 g cm^−3^). It is worth acknowledging that the processing conditions (such as humidity, processing temperature, shear stress, and time) might have influenced the mixing degree and consequently the observed morphology [21,22].

#### 3.1.2. Fourier Transform Infrared Spectroscopy

Figure S2a shows the FTIR spectra of SPEU, PHBV, and BG, displaying the characteristic bands of their functional groups. The analysis of SPEU has been previously reported by the authors [38,39]. The FTIR spectrum of PHBV exhibits a strong band at 1721 cm^−1^ associated with the stretching of the C=O bond (νC=O), corresponding to the characteristic ester group of PHA [21,42]. Additionally, the shoulder observed around 1740 cm^−1^ can be assigned to the stretching of the C=O group in the amorphous regions of PHBV [43]. The bands located in the region of 3020–2850 cm^−1^ correspond to the symmetric and asymmetric stretching modes of methyl and methylene groups in PHBV. Specifically, the bands within the ranges of 3020–2960 cm^−1^ (ν_a_ C-H in CH_3_), 2950–2900 cm^−1^ (ν_a_ C-H in CH_2_), and 2900–2850 cm^−1^ (ν_s_ C-H in CH_3_) are observed as five overlapped bands at approximately 3009, 2996, 2975, 2933, and 2875 cm^−1^ [44]. Moreover, the bands in the 1500–800 cm^−1^ region may be associated with H-C-H bending vibrations from CH_3_ and CH_2_, as well as the stretching vibrations of C-O-C and C-C. The overlapping of bands in this region of the spectrum prevents a direct and straightforward assignment [21,42], and its complete elucidation was not of major interest in this work. The most significant differences between both polymers are observed in the carbonyl group region (νC=O). Moreover, the bands corresponding to carbonyl-urea amide II and III groups linked by hydrogen bonding (νC-N and δN-H) are characteristic of SPEU. The spectrum of BG exhibits a broad band with two overlapping peaks in the range of 900–1100 cm^−1^, attributable to vibrations corresponding to the Si-O-Si and P=O of phosphate groups [3,21]. Additionally, Figure S2b presents the overlaid spectra of composite filaments comprising SPEU with PHBV and BG, normalized at 2933 cm^−1^, which corresponds to the asymmetric methylene stretching band (ν_a_C-H). The spectra exhibit remarkable similarity due to SPEU being the predominant component, with any minor distinctions attributed to the characteristic bands of PHBV and BG.

#### 3.1.3. Thermal Analysis

To evaluate the thermal properties of the materials, a series of three scans was performed: an initial heating step to eliminate the thermal history, a subsequent cooling to determine the crystallization capacity, and, finally, a second heating scan. Table 1 summarizes the thermal properties of PHBV powder and filaments based on SPEU, PHBV, and BG, while the DSC thermograms are presented in Figures S3 and S4.

PHBV exhibited narrow endothermic peaks at the same temperature in both heating scans (170–174 °C), associated with the melting of the crystalline regions. Notably, this temperature range overlaps with the melting peak of the HS of SPEU. The recrystallization of PHBV was observed around 87 °C.

In the case of SPEU-PHBV, two endothermic peaks were recorded for both heating runs (Figure S4a). The first one can be attributed to the melting of the SS of SPEU, while the second corresponds to the combined melting processes of PHBV and the HS of SPEU. This correlates not only with the melting temperatures of each polymer but also with the sum of their melting enthalpies. Additionally, three crystallization peaks were observed, which can be attributed to, in descending temperature order, the HS of SPEU (1), PHBV (2), and the SS of SPEU (3). These results suggest a low interaction between the two polymers, consistent with observations from the SEM micrographs. Nevertheless, PHBV could act as a mechanical reinforcement for SPEU.

Filaments incorporating BG in their formulation exhibited some differences compared with the polymer blend after the first heating (Figure S4b). This outcome suggests that the presence of BG particles affects the interaction between both polymers after fusion in the initial heating. Consequently, during the cooling and second heating stages, only two peaks associated with recrystallization and melting, respectively, were observed. Additionally, there was a 50% decrease in the enthalpies of recrystallization and second melting associated with the HS and PHBV peaks. This decrease may be attributed to BG hindering the recrystallization of HS and PHBV, or alternatively, only one of the materials being able to recrystallize under these conditions (Table 1). While the differences between the first and second heating are noticeable, the first event represents the most significant thermal event when defining the conditions for the printing process.

**Figure 3:**
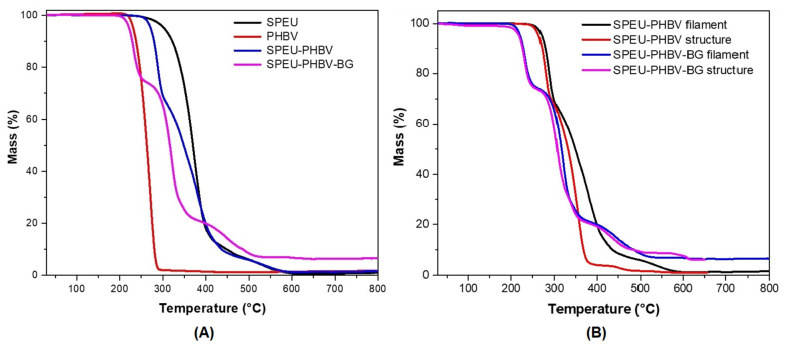
TGA curves of (**A**) PHBV, SPEU, and composite filaments; and (**B**) printed structures compared with their respective filaments.

Thermogravimetric analysis was conducted in an air atmosphere under both dynamic conditions (Figure 3a and Figure S5) and isothermal conditions (Figure S6). In the latter case, the test was performed for 60 min at a temperature of 185 °C, which is higher than the extrusion temperature but close to the FDM processing temperature. The thermograms show complete mass loss upon reaching 600 °C, except for the BG-containing sample, where the residue roughly corresponds to the percentage of BG incorporated into the mixture. Concerning SPEU, two main degradation stages are evident at 260–410 °C and 480–520 °C, associated with the thermal decomposition of hard and soft domains, respectively [38]. PHBV exhibits an onset decomposition temperature of 220 °C and reaches complete mass loss at approximately 285–290 °C, resulting in lower temperatures compared with SPEU. Regarding the SPEU-PHBV filament, it is apparent that the mass loss corresponds to the degradation of each polymer individually, as well as their respective content in the mixture, with only a slight peak shift, particularly notable for PHBV. This further suggests that PHBV could serve as a reinforcement for SPEU and supports the findings indicating the lack of miscibility between the two polymers. PCL-PHBV and PLA-PHBV systems developed by other research groups have exhibited similar thermal behavior [45,46]. On the other hand, the SPEU-PHBV-BG filaments displayed a different thermal behavior. The degradation started at a lower temperature, indicating that BG interacts with the polymers, causing a marked decrease in the thermal stability of both compounds.

Additionally, isothermal tests were conducted at 190 °C to gather relevant information for FDM processing. The SPEU filament recorded a mass loss of less than 1 wt%, whereas the SPEU-PHBV filament exhibited a 10 wt% loss. Although the pure filament demonstrates greater stability, the heating time in FDM processing is brief. Therefore, it can be inferred that the thermal degradation of the composite filaments during the printing process of a structure is minimal.

#### 3.1.4. Mechanical Behavior

The SPEU-PHBV filament displayed a Young’s modulus 146% higher than that of the SPEU filament, and the further addition of 5% BG resulted in a 6% increment compared with the blend (Table 2 and Figure S7a). This enhanced stiffness implies that both PHBV (E = 1.6 GPa [21]) and BG (E = 35 GPa [47]) serve as mechanical reinforcements for SPEU, indicating a significant increase in stiffness. This enhancement suggests that the addition of PHBV, with a Young’s modulus of 1.6 GPa [21], contributed to the overall rigidity of the composite. When 5% bioglass (BG) was further incorporated into the SPEU-PHBV blend, a modest but notable 6% increase in Young’s modulus was observed (Table 2 and Figure S7a). Given that bioglass has a much higher Young’s modulus (35 GPa) [47], even a small amount added to the polymer matrix can serve as an effective reinforcement, enhancing the composite stiffness. This finding aligns with SEM micrographs and thermal analyses, which suggest the immiscibility of the polymers and partial miscibility following the addition of BG.

The tensile strength at 5% strain (σ_5_) exhibited a similar trend. The SPEU-PHBV filament showed a considerable increase (75%) compared with the SPEU filament. However, the inclusion of BG led to a slightly lower value, which could be associated with a limited interaction between BG and the polymeric matrix.

### 3.2. Additive Manufacturing by FDM

Processing temperatures were set within the ranges of 183–185 °C for SPEU-PHBV and 190–191 °C for SPEU-PHBV-BG. The molten filaments were then deposited onto a preheated platform at 60 °C to promote good adhesion of the first layer and achieve the desired resolution for the final printed object. Fine-tuning this parameter was essential due to the inherent rigidity of PHBV. Printing operations were conducted at room temperature (approximately 21 °C), with the computer-controlled printing head operating at speeds ranging from 20 to 45 mm∙s^−1^.

**Figure 4:**
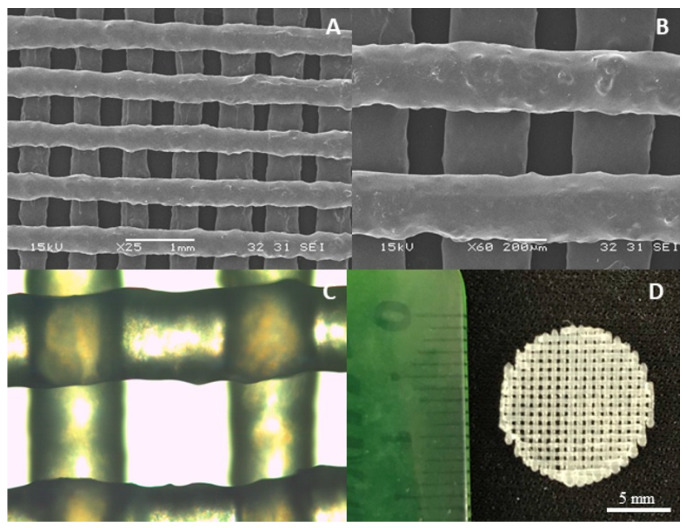
Printed structures of SPEU-PHBV (two layers). (**A**,**B**) SEM micrographs (25×, 60×), (**C**) optical microscope image (10×), and (**D**) image of the 3D-printed structure (top view, 13 mm diameter).

**Figure 5:**
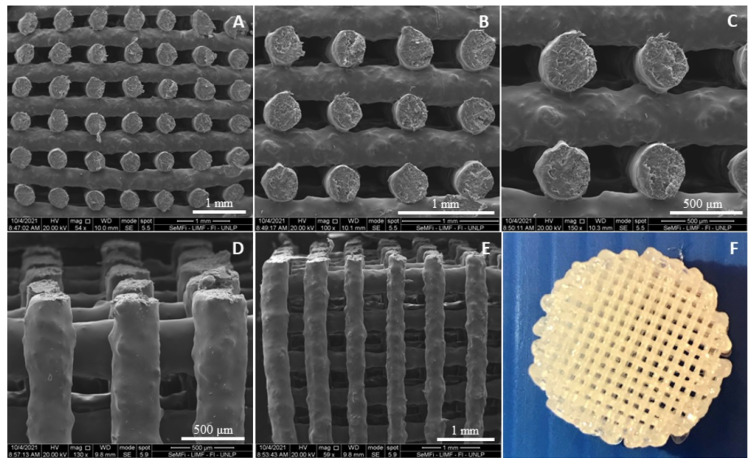
Printed structures of SPEU-PHBV (21 layers). SEM micrographs: (**A**–**C**) cross-sectional view (55×, 100×, and 150×), (**D**,**E**) top view (130×, 60×), and (**F**) image of the 3D-printed structure (top view, 15 mm diameter).

Figure 4 and Figure 5 display the 3D matrices obtained from SPEU-PHBV. As demonstrated by SEM micrographs of the top view and cross-sectional view of the 3D structures, the obtained pieces exhibited good resolution between printed lines. Kosorn and colleagues reported the successful additive manufacturing of filaments with varying PHBV/PCL ratios (with PCL similar to the soft segments of SPEU) by FDM, achieving a comparable level of definition [25,26].

**Figure 6:**
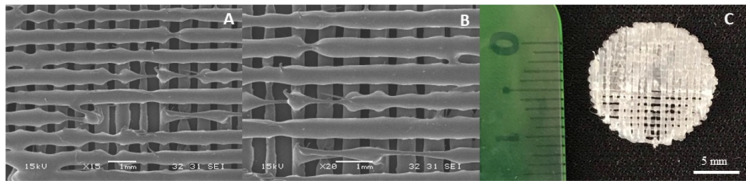
Printed structures of SPEU-PHBV-BG (two layers). (**A**,**B**) SEM micrographs (15×, 20×) and (**C**) image of the 3D-printed structure (top view, 13 mm diameter).

However, the introduction of BG complicated the printing process, leading to nozzle obstruction and disruptions in the flow of molten material. Despite adjustments made to processing parameters (such as printing speed, nozzle temperature, and filament diameter settings), achieving defect-free 3D structures proved to be challenging. To address this, one potential solution is to use a slightly larger nozzle diameter. This modification can help prevent clogging and promote a smoother flow of BG-enhanced filaments, reducing obstructions and improving the consistency of printed structures. As depicted in Figure 6, filament size, pore size, and the resulting porosity exhibited non-uniformity, underscoring the impact of BG interaction with the polymeric blend on the FDM printing process.

#### 3.2.1. Morphological Characterization and Contact Angle Measurements

Table 3 summarizes the morphological properties and contact angles (Figure S8) of the 3D-printed matrices. As expected, the addition of PHBV to SPEU led to an increase in the contact angle, indicating that the structures are more hydrophobic [48]. Furthermore, the incorporation of BG into the blend introduced a slight but non-significant decrease, aligning with the observation that the particles are mainly located inside the filaments. Regarding filament diameter, pore size, and apparent porosity (P_app_), the printed structures conformed to the parameters outlined in the CAD design, with the exception of the SPEU-PHBV-BG matrices, where defects were observed.

#### 3.2.2. Thermal Analysis

DSC thermograms were performed for the obtained 3D-printed samples, and the detailed results for all measured parameters are provided in Table 4. The structures exhibited a behavior similar to that obtained for their respective filaments, except for SPEU-PHBV, which exhibited a small exothermic peak during the second heating (Figure S9). This event could be attributed to the crystallization of a small fraction of PHBV. Liu and colleagues proposed that the thermal degradation of PHBV occurs above 160 °C through a mechanism involving a six-membered ring transition state [42]. Additionally, the FDM processing of these composite filaments requires temperatures exceeding 180 °C. Despite the brief dwell time of the filament in the extrusion head, Figure 3b illustrates that the 3D structure of SPEU-PHBV exhibits an onset of degradation at a lower temperature than the filament. This result can again be correlated with the immiscibility between both polymers and the behavior of pure PHBV. However, the situation differs for the SPEU-PHBV-BG material, for which no differences were recorded in the curves of their respective thermograms.

#### 3.2.3. Mechanical Behavior

The compression tests revealed the typical response expected from porous materials: an initial elastic regime followed by pore collapse and structure densification (Figure S10). Nevertheless, significant differences in the compression modulus of the composite matrices were observed, as indicated in Table 2 and Figure S7b. The obtained values revealed that the 3D structures of SPEU-PHBV exhibited a 246% higher stiffness than pure SPEU structures. Additionally, the addition of 5% BG to the polymer blend increased the compression modulus by 15.5%. Consequently, the incorporation of PHBV and BG had a positive impact on the Young’s modulus and compression strength of the printed matrices.

While the primary aim of designing 3D-printed structures with composite materials was focused on applications in bone tissue engineering, the compression stress measured was comparable to that of human proximal tibia trabecular bone [14,49]. Therefore, based on the mechanical evaluation conducted, it can be inferred that the bioresorbable elastomeric matrices of SPEU-PHBV-BG could also be suitable candidates for tissue regeneration of cartilage (E = 0.7–15.3 MPa) [2], articular cartilage (E = 0.3–0.8 MPa) [50], the meniscus (E = 0.09–0.29 MPa) [51], and the spinal cord (E = 0.04–0.06 MPa) [52].

## 4. Conclusions

Bioresorbable filaments of SPEU-PHBV and SPEU-PHBV-BG were successfully produced with no need for additives in their formulation. Likewise, the melting and solidification of filaments could be achieved by FDM without significantly inducing thermal degradation processes. However, while the production of 3D structures based on SPEU-PHBV with excellent dimensional precision was possible, it was not feasible for SPEU-PHBV-BG, where matrices showed defects and challenging reproducibility. The introduction of BG complicated the printing process, leading to nozzle obstruction and disruptions in the flow of molten material. A potential solution could be the use of a slightly larger nozzle diameter to help prevent clogging and promote a smoother flow of BG-enhanced filaments, reducing obstructions and improving the consistency of printed structures. Despite these obstacles, the physicochemical, thermal, and mechanical properties of these materials hold promise. The incorporation of Bioglass 45S5 had a minor impact on the hydrophilicity of the scaffolds but acted as a reinforcement, enhancing both the elastic modulus of the filaments and the compression mechanical properties of the 3D matrices. While the aim of designing 3D-printed structures with composite materials was geared toward bone tissue engineering applications, the compression stress exhibited by these matrices was found to be optimal for tissues such as human proximal tibial trabecular bone. Therefore, these materials could potentially be used in the design and manufacture of customized structures for applications in both hard and soft tissue engineering. This is particularly significant in areas where printable bioresorbable materials are lacking. Finally, in vitro experiments are in progress, and the results will be published elsewhere.

## Figures and Tables

**Table 1:** Thermal properties of PHBV and filaments, as determined by DSC.

Material	T_m_^a^ (°C)	ΔH_m_^a^ (J g^−1^)	T_m_^b,c^ (°C)	ΔH_m_^b,c^ (J g^−1^)		
			First heating			
PHBV powder			173.6	89.0		
SPEU	11.4	4.8	174.1	42.7		
SPEU-PHBV	12.8	3.6	176.1	53.9		
SPEU-PHBV-BG	12.8	2.5	173.9	51.1		
			Cooling			
	T_c_^a^ (°C)	ΔH_c_^a^ (J g^−1^)	T_c_^b^ (°C)	ΔH_c_^b^ (J g^−1^)	T_c_^c^ (°C)	ΔH_c_^c^ (°C)
PHBV powder					87.0	−72.0
SPEU	−11.8	−4.7	148.4	−42.6		-
SPEU-PHBV	−12.7	−3.5	149.8	−32.2	72.1	−16.0
SPEU-PHBV-BG	−25.5	−2.5	132.3	−25.5		
			Second heating			
	T_m_^a^ (°C)	ΔH_m_^a^ (J g^−1^)	T_m_^b,c^ (°C)	ΔH_m_^b,c^ (J g^−1^)		
PHBV powder			170.6	84.2		
SPEU	19.3	5.2	171.2	40.4		
SPEU-PHBV	20.6	3.7	174.6	50.1		
SPEU-PHBV-BG	19.2	10.2	153.9	20.1		

^a^ Values associated with SS of SPEU. ^b^ Values associated with HS of SPEU. ^c^ Values associated with PHBV.

**Table 2:** Mechanical properties of filaments and printed structures.

Material	Filament		3D-Printed Structure	
	E ^1^ (MPa)	σ_5_ (MPa)	E ^2^ (MPa)	σ_10_ (MPa)
SPEU	145 ± 18	5.9 ± 0.3	0.13 ± 0.03	1.33 ± 0.08
SPEU-PHBV	357 ± 38	10.4 ± 0.4	0.45 ± 0.02	1.37 ± 0.14
SPEU-PHBV-BG	379 ± 30	9.4 ± 0.2	0.53 ± 0.05	1.48 ± 0.13

^1^ Determined from the slope of the stress-strain curves at 1% strain. ^2^ Determined from the slope of the stress-strain curves at 0.2–1.2% strain.

**Table 3:** Morphological parameters and contact angles of the printed structures.

Filament Material	Diameter (µm)	Pore Size (µm)	P_app_ (%)	Contact Angle
SPEU	388 ± 29	328 ± 30	55.2 ± 3.9	95.6 ± 2.1°
SPEU-PHBV SPEU-PHBV-BG	399 ± 18 -	339 ± 50 -	50.4 ± 2.5 -	110.7 ± 1.3° 105.8 ± 2.0°

**Table 4:** Thermal properties determined by DSC (first heating, cooling, and second heating) for the 3D-printed structures.

Material	T_m_^a^ (°C)	ΔH_m_^a^ (J g^−1^)	T_m_^b^ (°C)	ΔH_m_^b^ (J g^−1^)		
			First heating			
SPEU	16.0	10.3	173.7	43.8		
SPEU-PHBV	15.5	4.1	172.4	52.4		
SPEU-PHBV-BG	13.7	4.6	171.2	45.8		
			Cooling			
	T_c_^a^ (°C)	ΔH_c_^a^ (J g^−1^)	T_c_^b^ (°C)	ΔH_c_^b^ (J g^−1^)	T_c_^c^ (°C)	ΔH_c_^c^ (°C)
SPEU	−11.6	−9.1	150.5	−42.2		
SPEU-PHBV	−12.5	−4.1	151.1	−32.6	65.9	−11.6
SPEU-PHBV-BG	−26.5	−5.01	133.0	−31.6		
			Second heating			
	T_m_^a^ (°C)	ΔH_m_^a^ (J g^−1^)	T_m_^b^ (°C)	ΔH_m_^b^ (J g^−1^)	T_c_^c^ (°C)	ΔH_c_^c^ (°C)
SPEU	21.6	10.2	171.1	43.4		
SPEU-PHBV	21.4	4.4	172.2	50.7	46.9	3.4
SPEU-PHBV-BG	18.4	9.0	152.9	24.1		

^a^ SPEU soft segments. ^b^ In filaments containing only SPEU, the values are associated with hard segments of SPEU. ^c^ Values associated with PHBV.

## Data Availability

The original contributions presented in the study are included in the article/Supplementary Material; further inquiries can be directed to the corresponding author/s.

## Supplementary Materials

The following supporting information can be downloaded at: https://www.mdpi.com/article/10.3390/polym16233355/s1, Figure S1: SEM micrographs of the cross-sectional views of the filaments: SPEU-PHBV (A), and SPEU-PHBV-BG (B, C) (7500X and 24000X, respectively); Figure S2: (A) FTIR spectra of SPEU, PHBV, and BG raw materials; (B) normalized FTIR spectra for SPEU-based filaments and BG; Figure S3: DSC thermograms (first heating, cooling, and second heating) for (A) SPEU and (B) PHBV; Figure S4: DSC thermograms (first heating, cooling, and second heating) for the filaments: (A) SPEU-PHBV and (B) SPEU-PHBV-BG; Figure S5: Thermal stability study for pure and composite systems under dynamic conditions: Derivative TGA curves; Figure S6: Isothermal study at 185 °C of thermal stability for SPEU and SPEU-PHBV; Figure S7: Young’s modulus values for (A) Filaments (mean ± SD, n = 5); (B) 3D-printed scaffolds (mean ± SD, n = 3). One-way ANOVA with Tukey post-hoc test for comparisons among normally distributed groups. Differences were considered significant at * *p* < 0.05; Figure S8: Water contact angle images for: (A) SPEU, (B) SPEU-PHBV, and (C) SPEU-PHBV-BG; Figure S9: DSC thermograms for SPEU-PHBV-BG 3D-printed structures (first heating, cooling, and second heating); Figure S10: Representative stress-strain curves in compression for SPEU, SPEU-PHBV, and SPEU-PHBV-BG structures. Insert: curves in both cases for strains up to 40%.
