# Supplementary material for: 3D-Printed Poly(ester urethane)/Poly(3-hydroxybutyrate-co-3-hydroxyvalerate)/Bioglass Scaffolds for Tissue Engineering Applications
Source: Polymers (Basel). 2024 Nov 29;16(23):3355. doi: 10.3390/polym16233355 (PMC11644432; doi:10.3390/polym16233355)
Supplement: Supplementary file 1 [file polymers-16-03355-s001.zip › polymers-3291964-supplementary.pdf]

2. MATERIALS AND METHODS

2.4. Filament and printed structures characterization

Fourier transform infrared analyses were performed using a Mattson Genesis II FTIR spectrometer equipped with an attenuated total reflectance (ATR) accessory. Spectra were recorded from 64 scans over a frequency range of 4000–400 cm^{-1} at a resolution of 2 cm^{-1}. Normalization was performed by the methylene stretching bands (ν_aC-H and ν_sC-H, 2937 and 2864 cm^{-1}, respectively).

Differential scanning calorimetry (DSC) was carried out in a Pyris 1 calorimeter (Perkin-Elmer) with an intracooler accessory. Heating/cooling/heating cycles were conducted in nitrogen atmosphere in the range of −60 to 200°C at a heating rate of 10°C.min^{-1} and a cooling rate of 5°C.min^{-1}.

Thermogravimetric analyses (TGA) were performed in a TGA-50 (Shimadzu, Japan). Samples were evaluated under dynamic and isothermal conditions in air atmosphere. For the dynamic study, heating was performed from room temperature to 800°C at a rate of 10°C.min^{-1}, and the initial degradation temperature was determined from the onset of the mass loss curve. For the isothermal study, the temperature was raised at a rate of 10°C.min^{-1} and then kept constant at 190°C, recording the mass for 1.5 h.

Water contact angles were measured at room temperature with a goniometer (ramé-hart Instrument Co.) using the sessile drop method. A droplet of milli-Q water (5 μL) was deposited onto the surface of a 3D-printed non-porous sample and after 1 minute the contact angle was measured. The values presented are the arithmetic mean of six measurements (±SD).

The surface morphology of filaments and printed structures was examined by scanning electron microscopy (SEM) using a Jeol JSM-6460LV microscope (JEOL Ltd., Japan) after gold sputtering. To assess BG microparticle distribution inside the filaments, fracture surfaces were obtained by immersion of the filaments in liquid nitrogen and subsequent cutting with a scalpel. SEM micrographs were analyzed using an image processing software (Image Pro Plus; Media Cybernetics Inc.). The mean strut diameter and in-plane pore size were determined from 12 randomly selected measurements per sample to obtain a meaningful statistical value. Images of 3D-printed structures were also obtained from a Leica DMLB Optical Microscope (Danaher Corp.). The dimensional accuracy was determined from cylindrical open-pore structures (CAD model: diameter = 15 mm, height = 7.5 mm, pore size = 350 μm, strut diameter = 400 μm, overall porosity = 50%; n = 3). Sample height and diameter were measured with a Digimatic Caliper Series 573 (Mitutoyo Corp., Japan). The height was determined at four different points of the structure, while the diameter was measured on the X and Y axes at the cylinder edge and averaged. To determine the accuracy, the obtained dimensions were compared with the original CAD design. The apparent porosity (Papp) of the scaffolds was determined using the following equation [39]:

$$Papp\ (\%) = \left[1 - \frac{\delta_{matrix}}{\delta_{material}}\right] x\ 100$$

where δ_{matrix} is the density of the scaffold, as determined by measuring its mass and dimensions; and $\delta_{material}$ is the density of the corresponding filament.

Mechanical testing of the filaments was performed in a dynamic mechanical analyzer (DMA Q800, TA Instruments) to determine the Young's modulus under quasistatic conditions. Tensile tests were carried out on a strain ramp mode with a preload force of 0.01 N and a displacement rate of 1 mm.min^{-1}. The elastic modulus was calculated from the linear region of the stress–strain curves (1% strain). At least five measurements were taken and averaged for each sample.

Mechanical characterization of 3D-printed scaffolds was carried out by uniaxial compression tests using a universal testing machine (EMIC 23-50, Instron), according to ASTM D695 [40]. The samples were compressed at a rate of 1 mm.min^{-1} up to a deformation of 80% using a 30 kN load cell. The Young's modulus was determined from the slope of the stress-strain curves at a range of 0.2-1.2% strain.

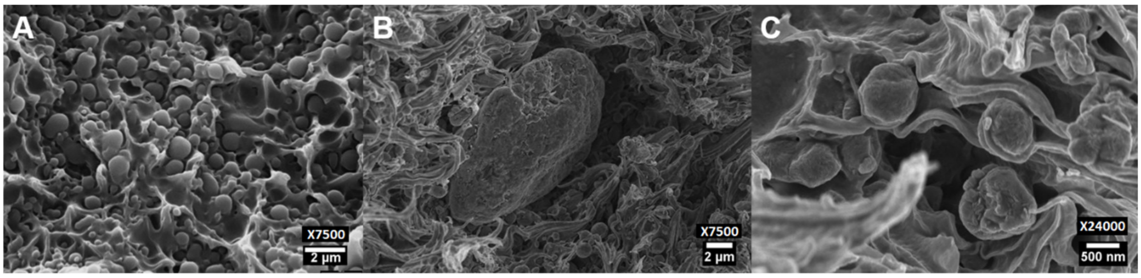

Figure S1. SEM micrographs of the cross-sectional views of the filaments: SPEU-PHBV **(A)**, and SPEU-PHBV-BG **(B, C)** (7500X and 24000X, respectively).

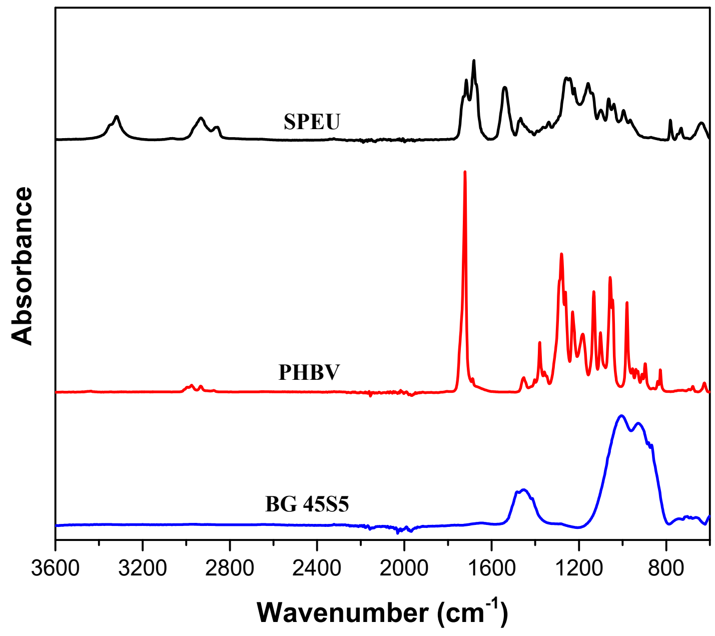

(A)

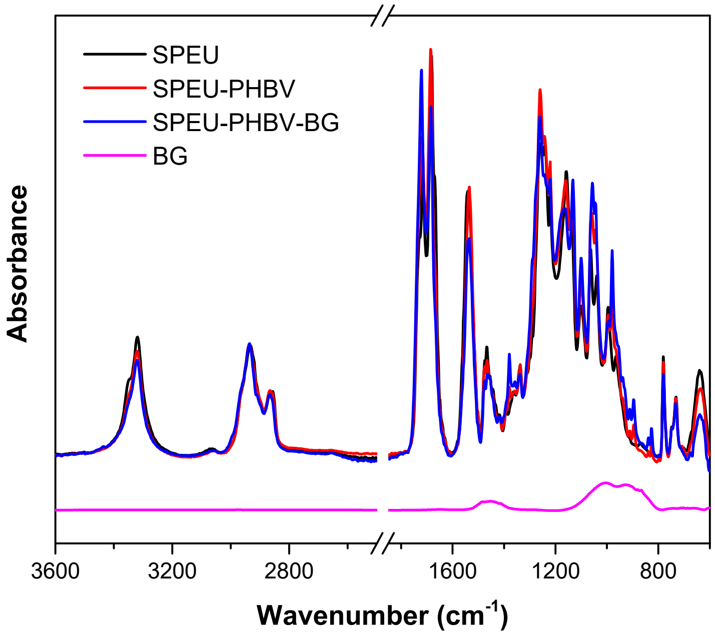

(B)

Figure S2. (A) FTIR spectra of SPEU, PHBV, and BG raw materials; (**B)** normalized FTIR spectra for SPEU-based filaments and BG.

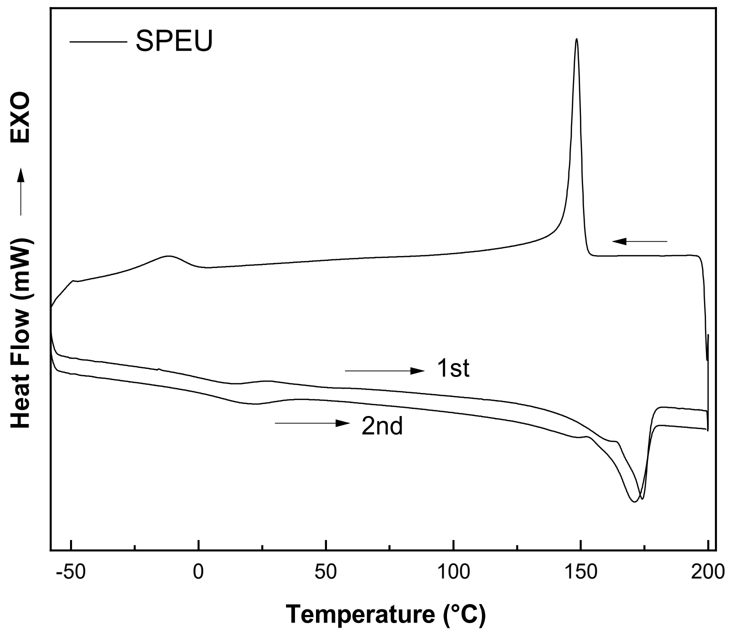

(A)

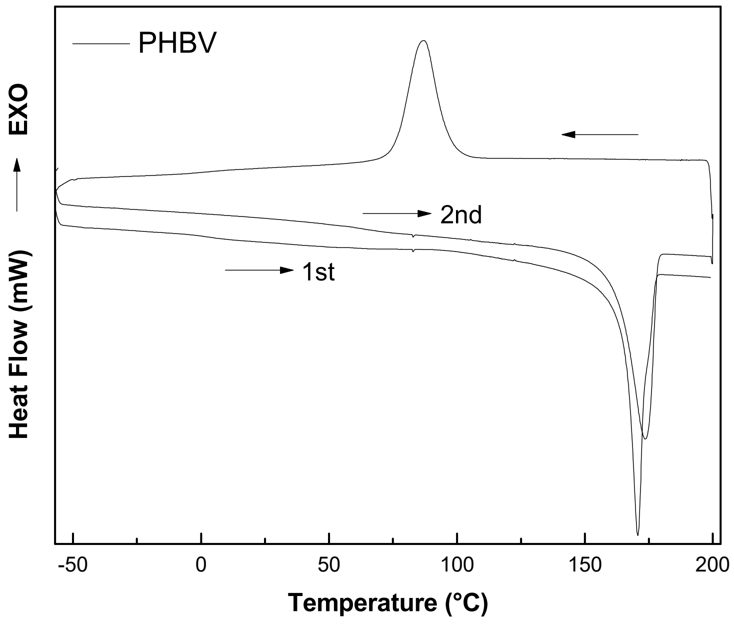

(B)

Figure S3. DSC thermograms (first heating, cooling, and second heating) for (**A**) SPEU and (**B**) PHBV.

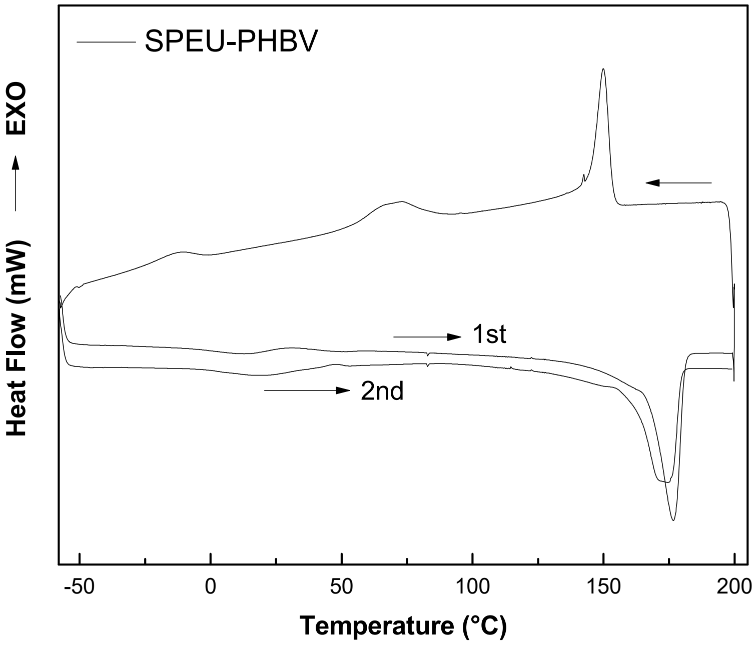

(A)

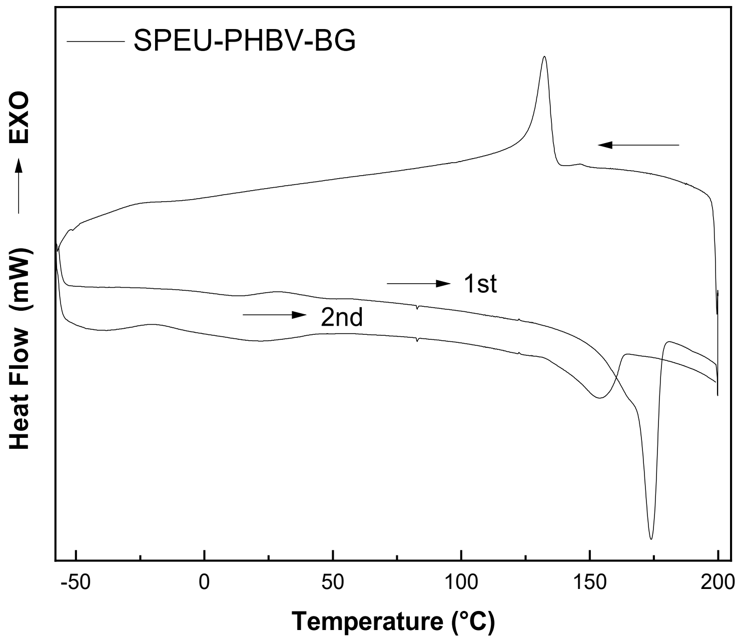

(B)

Figure S4. DSC thermograms (first heating, cooling, and second heating) for the filaments: (**A**) SPEU-PHBV and (**B**) SPEU-PHBV-BG.

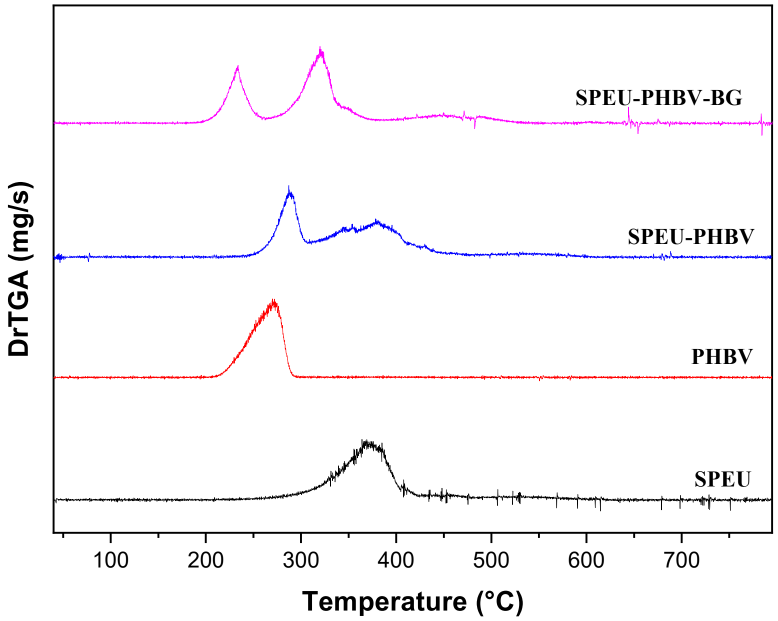

Figure S5. Thermal stability study for pure and composite systems under dynamic conditions: Derivative TGA curves.

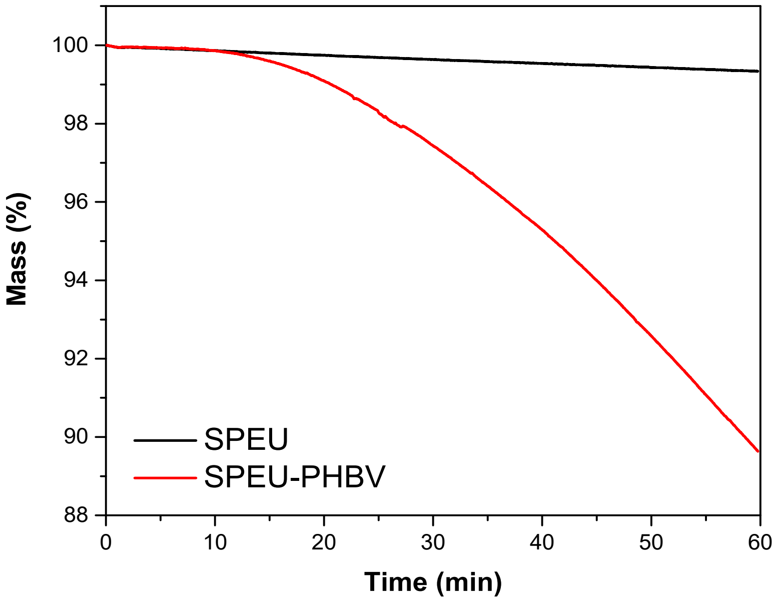

Figure S6. Isothermal study at 185 °C of thermal stability for SPEU and SPEU-PHBV.

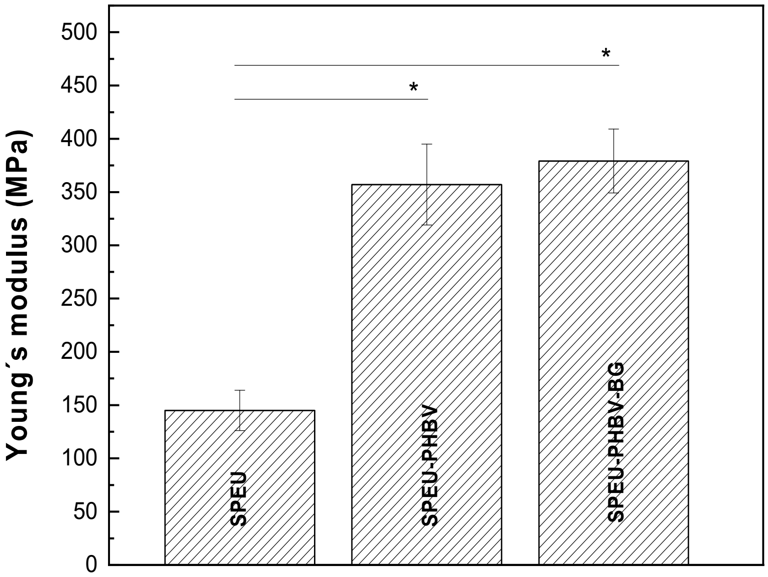

(A)

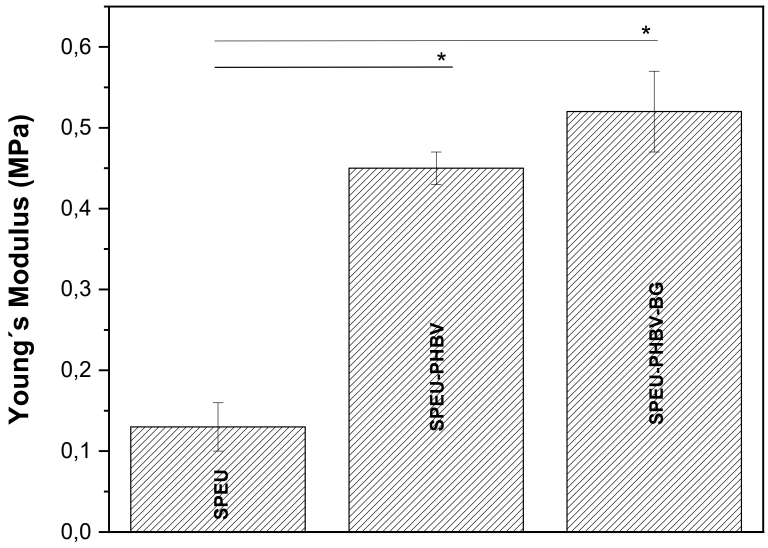

(B)

Figure S7. Young's modulus values for (**A**) Filaments (mean ± SD, n = 5); (**B**) 3D-printed scaffolds (mean ± SD, n = 3). One-way ANOVA with Tukey post-hoc test for comparisons among normally distributed groups. Differences were considered significant at $*p < 0.05$.

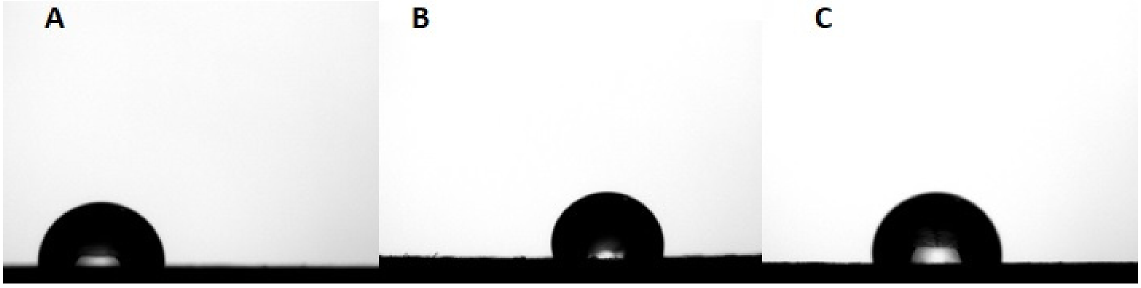

Figure S8. Water contact angle images for: (**A**) SPEU, (**B**) SPEU-PHBV, and (**C**) SPEU-PHBV-BG.

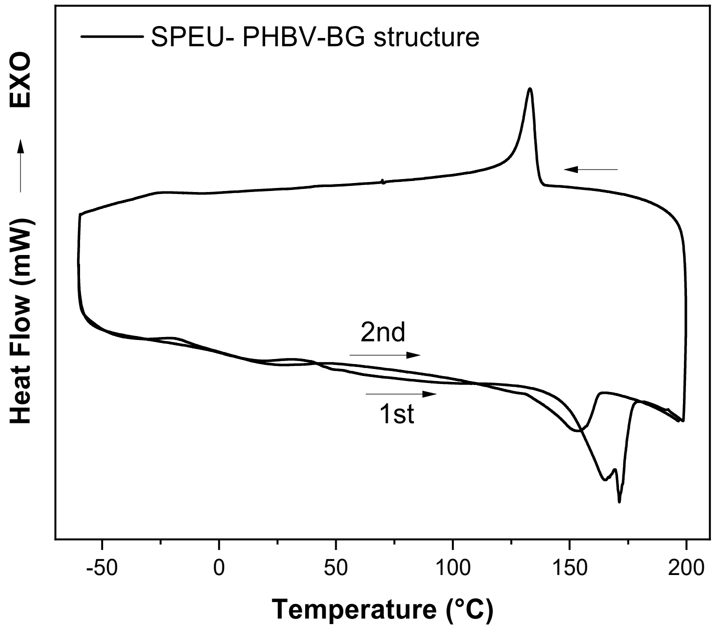

Figure S9. DSC thermograms for SPEU-PHBV-BG 3D-printed structures (first heating, cooling, and second heating).

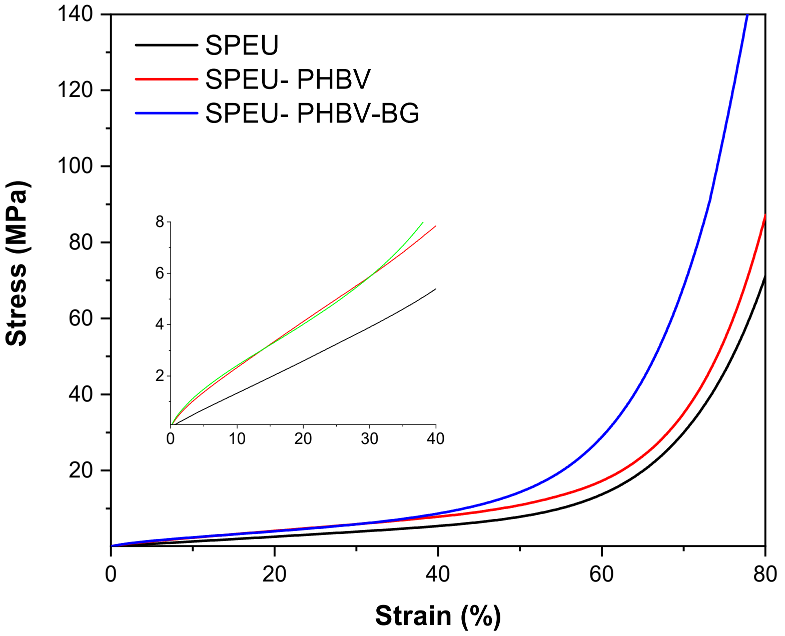

Figure S10. Representative stress-strain curves in compression for SPEU, SPEU-PHBV, and SPEU-PHBV-BG structures. Insert: curves in both cases for strains up to 40%.
